# Resection and reconstruction for radial polydactyly Type IV-D in 206 cases: a retrospective clinical analysis

**DOI:** 10.1186/s12891-022-05119-w

**Published:** 2022-02-22

**Authors:** Yuzhou Liu, Xiuyue Xu, Le Wang, Jie Lao, Yongqing Zhuang, Yousheng Fang

**Affiliations:** 1 Department of Hand Surgery, Huashan Hospital, Fudan University, Shanghai, People’s Republic of China; 2 Key Laboratory of Hand Reconstruction, Ministry of Health, Shanghai, People’s Republic of China; 3 Shanghai Key Laboratory of Peripheral Nerve and Microsurgery, Shanghai, China; 4 Department of Pediatric Surgery, Shanghai Jiao Tong University Medical School Affiliated Ruijin Hospital, Shanghai, People’s Republic of China; 5 Hand and Microvascular Surgery Department, Shenzhen People’s Hospital, Guangdong Province, 518020 China; 6 Department of Hand Surgery, Shanghai Huashan Hospital, 12 Wulumuqi Zhong Road, Jing An District, Shanghai, 200040 China

**Keywords:** Radial polydactyly, Wassel type, Resection, Reconstruction

## Abstract

**Background:**

Radial Polydactyly Type IV-D deformity is difficult to treat because of the most complex bone and soft tissue anomalies. Resection and reconstruction for one of the two thumbs was an option for treatment.

**Objective:**

The study was to present our method of resection and reconstruction with a new incision for radial polydactyly Type IV-D and evaluate the clinical efficacy comprehensively using Rotterdam assessment system in a large sample.

**Methods:**

206 cases of type IV-D thumb duplication underwent resection and reconstruction surgical treatment between 2010 and 2019. Two equal triangle flap incisions were designed around the radial thumb. The radial thumb was resected and the ulnar thumb was reconstructed in aspects of bone, tendons, ligaments and abductor pollicis brevis.

The clinical results were evaluated using Rotterdam assessment system.

**Results:**

The mean follow–up period was 2.2 years (SD 1.5). The mean age of the patients was 9 months (SD 1.8) at the time of operation. The mean ranges of active IP and MP joint flexion and extension were 110° and 26°. The mean angulations for IP and MP joint instabilities were 3° and 11°, relatively. Angulation for palmar abduction was 58°. The mean appearance domain score was 8.9. The average parental satisfaction score was 2.5 and the average patient-reported pain score was 2.1. The mean functional domain score for all patients was 6.6. The average appearance domain score was 8.9. The mean patient-reported domain score was 4.5. The mean Rotterdam outcome score was 20.0, equivalent to 67% of the full score. The postoperative score of patients over two years old was significantly lower than that of patients under two years old.

**Conclusion:**

Resection and reconstruction method with two equal triangle flap incisions was a recommended treatment for radial polydactyly Type IV-D.

**Level of evidence:**

IV

## Introduction

In China, thumb polydactyly is the most common congenital hand anomaly [9]. Wassel Type IV is the most common type in thumb duplication (polydactyly), accounting for about 50% [8], which involves duplication of the proximal phalanges at the metacarpophalangeal (MCP) joint [12]. In 1996, Hung et al. proposed subdividing type IV abnormalities into four subtypes: IV-A hypoplastic type (12%), IV-B ulnar deviated type (64%), IV-C divergent type (15%), and IV-D convergent type (9%) [8]. The Wassel type IV-D thumb duplication subtype involves two hypoplastic and symmetrical thumbs with valgus of the MCP joint and varus of the interphalangeal joint [7]. Type IV-D deformity is difficult to treat because of the most complex bone and soft tissue anomalies [6]. It is most likely to have residual deformities, such as zig-zag deformity [11]. Two surgical techniques are available for type IV-D reconstruction [1]: reconstructing one of the two thumbs and the Bilhaut-Cloquet procedure [2]. The traditional incision for reconstructing one of the two thumbs was a racket shape, which resulted in a linear scar on the radial dorsal side of the thumb. We designed a new incision to avoid a linear scar. There was no statistical report of resection and reconstruction for type IV-D thumb duplication with large sample size in China. In this study, we would like to present our method of resection and reconstruction for radial polydactyly Type IV-D and evaluate the clinical results of 206 cases using a comprehensive scoring system retrospectively.

## Materials and methods

**Fig. 1:**
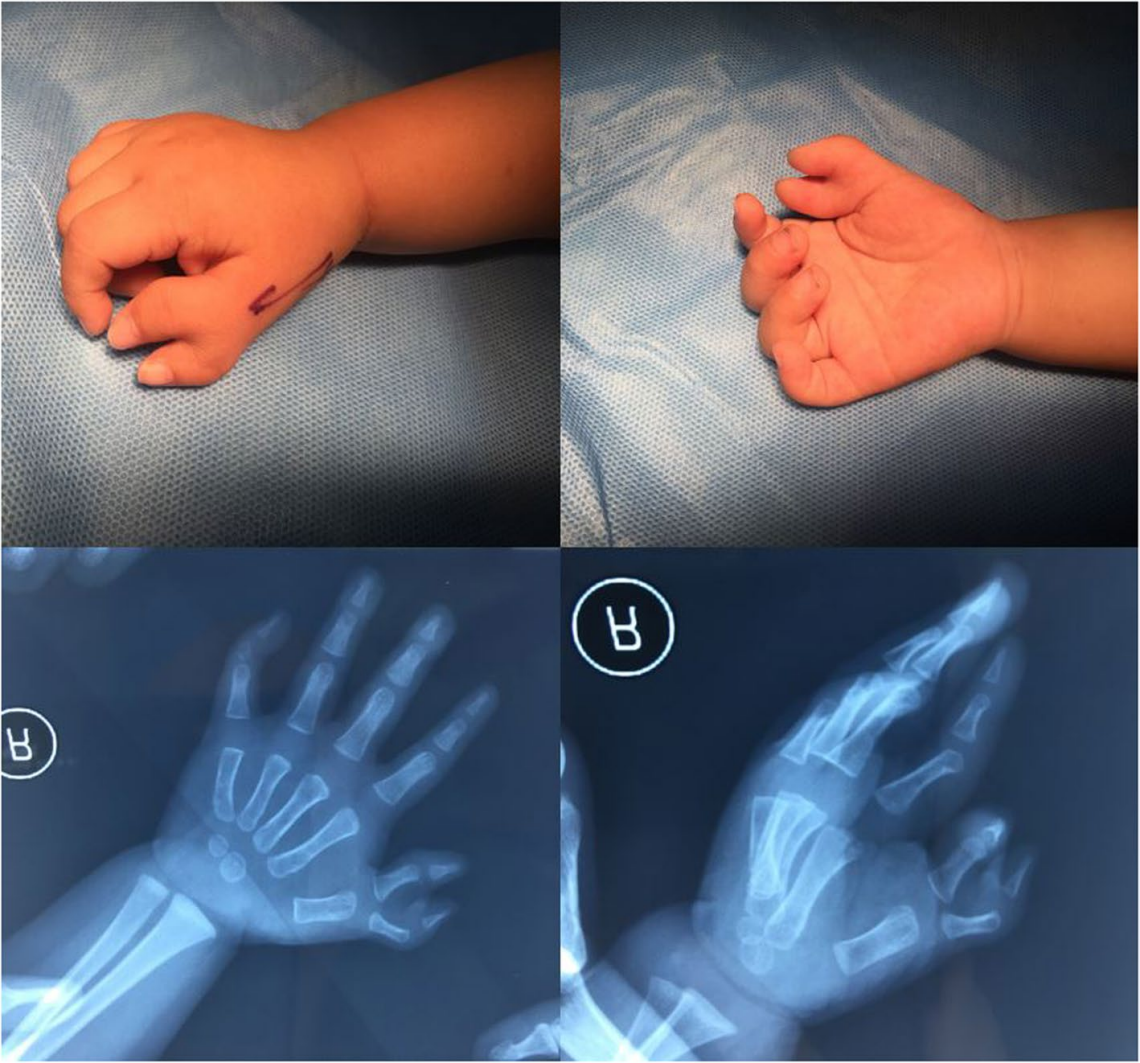
Wassel type IV-D thumb duplication

After institutional review board approval, a retrospective review of 206 cases of Wassel type IV-D thumb duplication (Fig. 1) in 173 patients who underwent surgical treatment between 2010 and 2019 was carried out.

Inclusion criteria: Patients were born with radial polydactyly types IV-D. The patients had radial thumb resection and ulnar thumb reconstruction in our institution.

Exclusion criteria: Patients had growth disturbance or additional congenital malformations of the upper extremity. Ulnar thumb resection or Bilhaut-Cloquet procedure was carried out as for radial polydactyly types IV-D.

The mean age at surgical procedure was 9 months (SD 1.8). In preoperative physical examination, the duplicated thumbs were found divergent at the MP joint and the distal phalanges of the duplicated thumbs were convergent at the interphalangeal (IP) joint. All parents or guardians of the patients were informed about the surgical methods, and written informed consent was obtained.

### Surgical technique

**Fig. 2:**
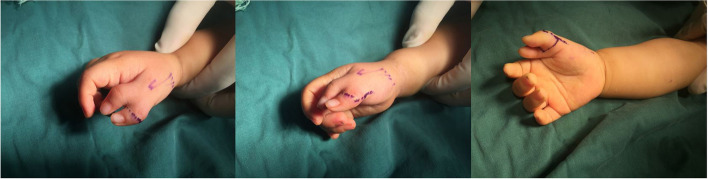
Two equal triangle flap incisions were designed around the radial thumb

Thumb size was assessed by comparing the affected thumb to the contralateral “normal” thumb. Ideally, the ulnar thumb was preserved because it was most commonly the larger thumb and maintained the critical ulnar collateral ligament at the MP joint, which was important for stability during pinch. We designed two equal triangle flap incisions (Fig. 2) around the smaller, more radial thumb. The volar incision was at the dermatoglyph of the thumb MP joint. The ulnar incision was in the center between two thumbs. The length of the radial incision was equal to that of the volar incision. The length of the dorsal incision was equal to that of the ulnar incision. The angle formed by the volar and ulnar incisions was equal to that formed by the dorsal and radial incisions.

#### Resection

The neurovascular bundles were identified. The nerves in the thumb to be excised were sharply incised and vessels were cauterized at the distal end of MP joint. The radial bundle for the maintained thumb was protected.

**Fig. 3:**
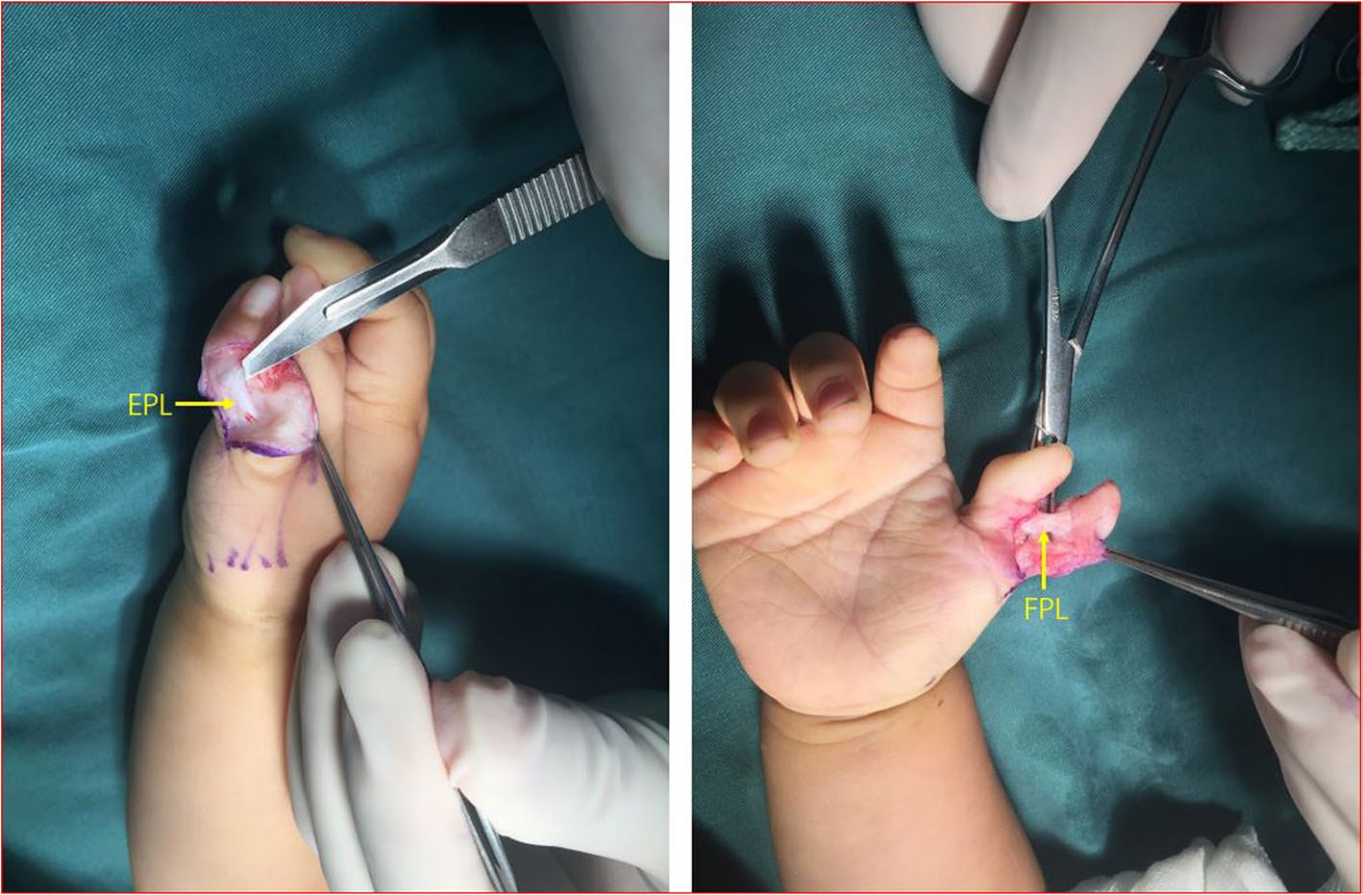
FPL (yellow arrow) and EPL (yellow arrow) were traced proximally to their bifurcation from the ulnar thumb’s tendons and were divided sharply

Flexor pollicis longus (FPL) and extensor pollicis longus (EPL) tendons were traced proximally to their bifurcation from the ulnar thumb’s tendons and were divided sharply (Fig. 3). The insertion of the flexor and extensor tendons on the base of distal phalanx of radial thumb were detached and preserved.

**Fig. 4:**
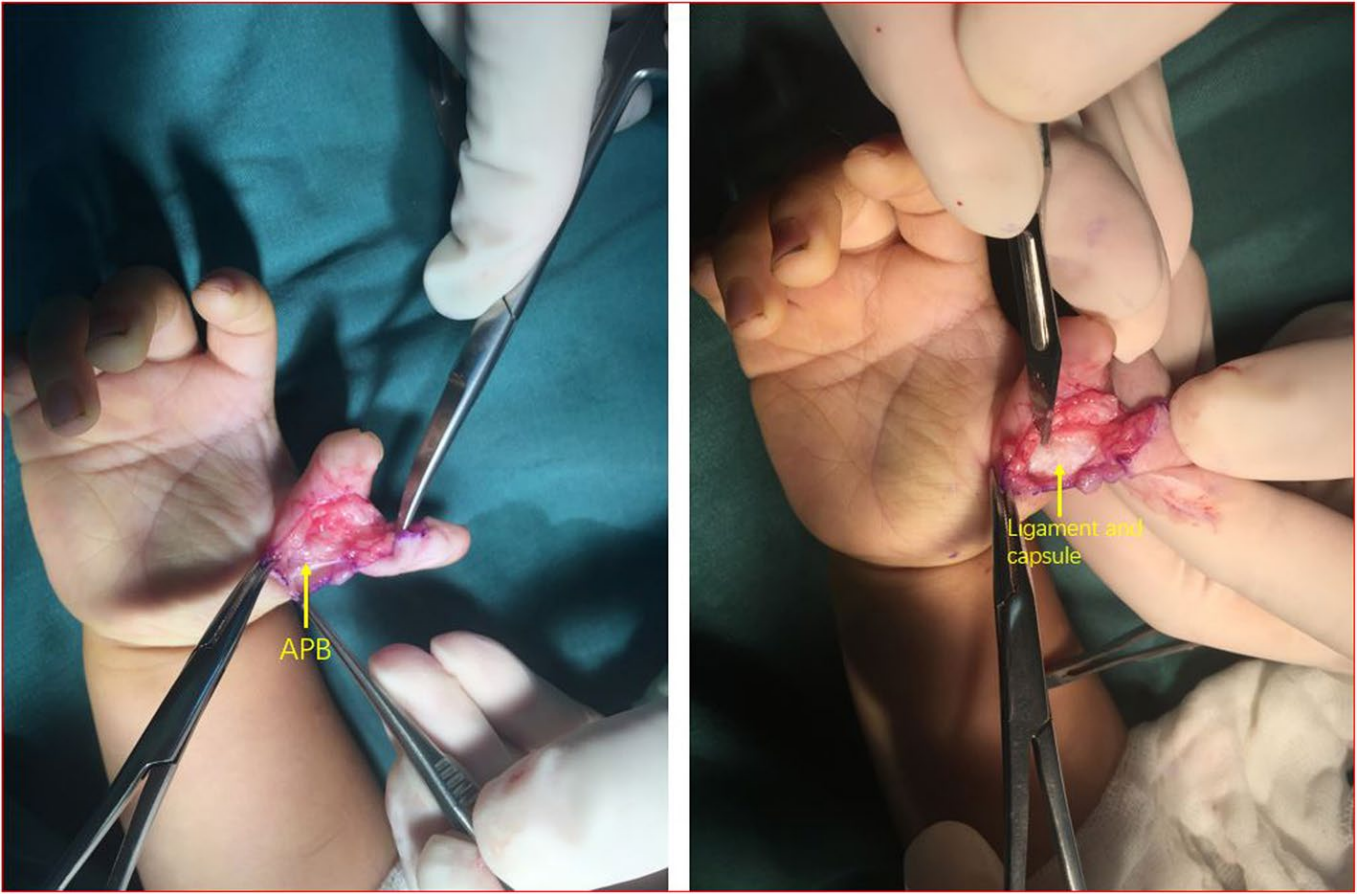
The insertion of APB (yellow arrow) and the radial collateral ligament (yellow arrow) of MP joint on the radial duplicated thumb were carefully detached and preserved

The insertion of the abductor pollicis brevis (APB) and the radial collateral ligament of MP joint on the radial duplicated thumb were carefully detached and preserved (Fig. 4).

The radial thumb was amputated at the metacarpophalangeal joint. The metacarpal head was then inspected. It was widened and had two facets, one for each phalanx.

#### Reconstruction

**Fig. 5:**
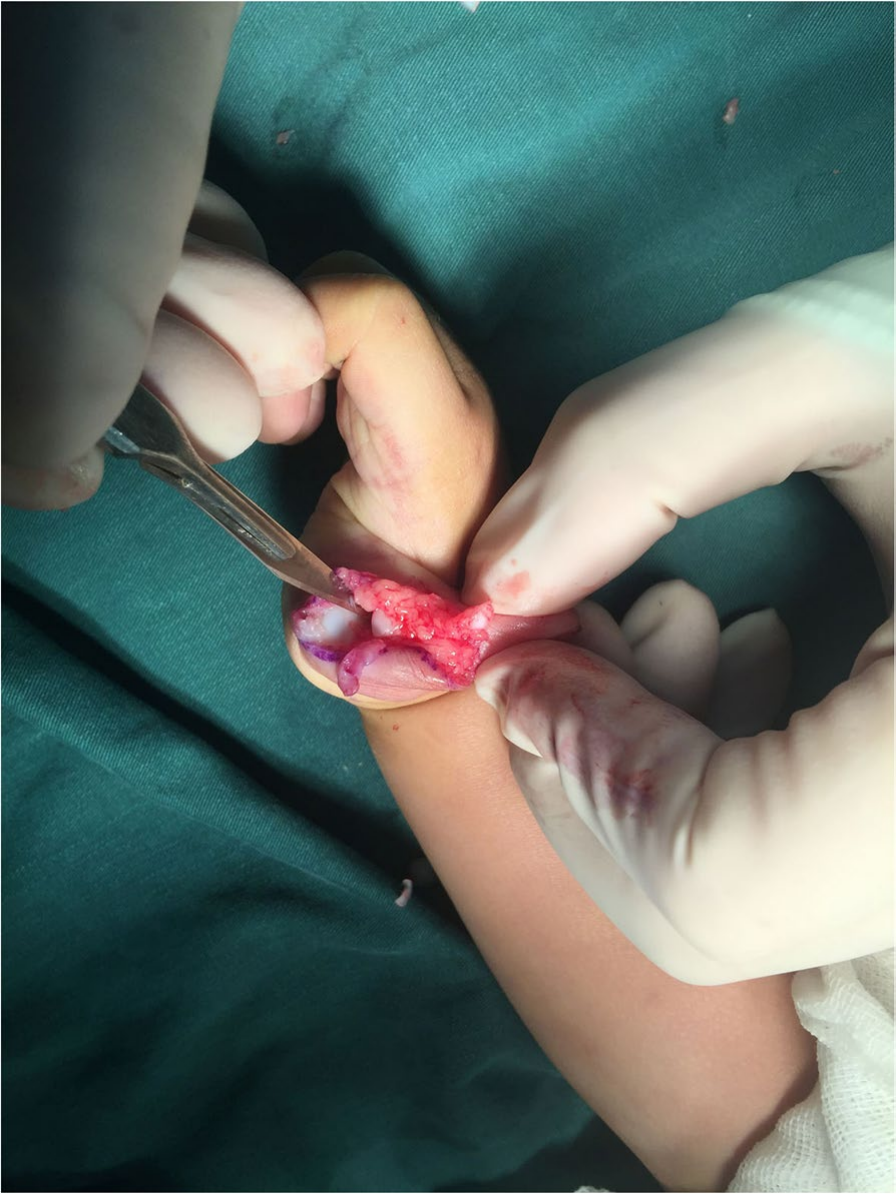
The wide metacarpal head was reshaped by cutting off the radial overhanging part

If the angular deformity was less than 25°, the wide metacarpal head was reshaped by cutting off the radial overhanging part (Fig. 5). If the angular deformity was greater than 25°, besides cutting off the radial overhanging part, we preferred a closing wedge osteotomy at the neck of the first MC and proximal phalanx. Then, the angulations at the MP and IP joints were realigned by reducing the proximal and distal phalanges along the longitudinal axis of metacarpal. The thumb was held in upright position by inserting a K wire through the IP and MP joints.

A longitudinal incision was made on the ulnar side of IP joint of the maintained thumb.

**Fig. 6:**
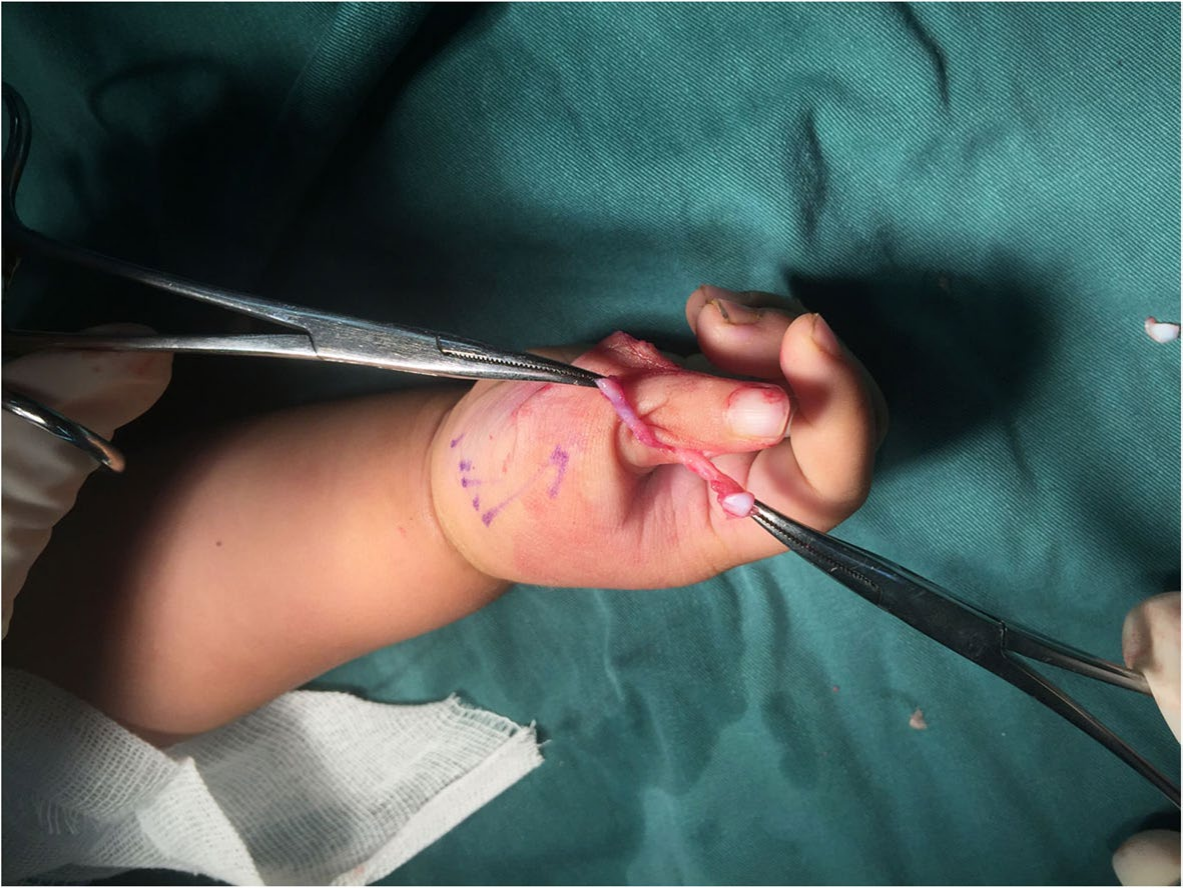
The transferred FPL and EPL tendons were tensioned

The ulnar collateral ligament of IP joint was tightened. The FPL and EPL tendons of the preserved radial thumb were transferred to the ulnar side of IP joint. The transferred FPL and EPL tendons were tensioned (Fig. 6). The insertions were repositioned by periosteal sutures with a 4–0 nonabsorbable suture at the ulnar side of the distal phalangeal base.

**Fig. 7:**
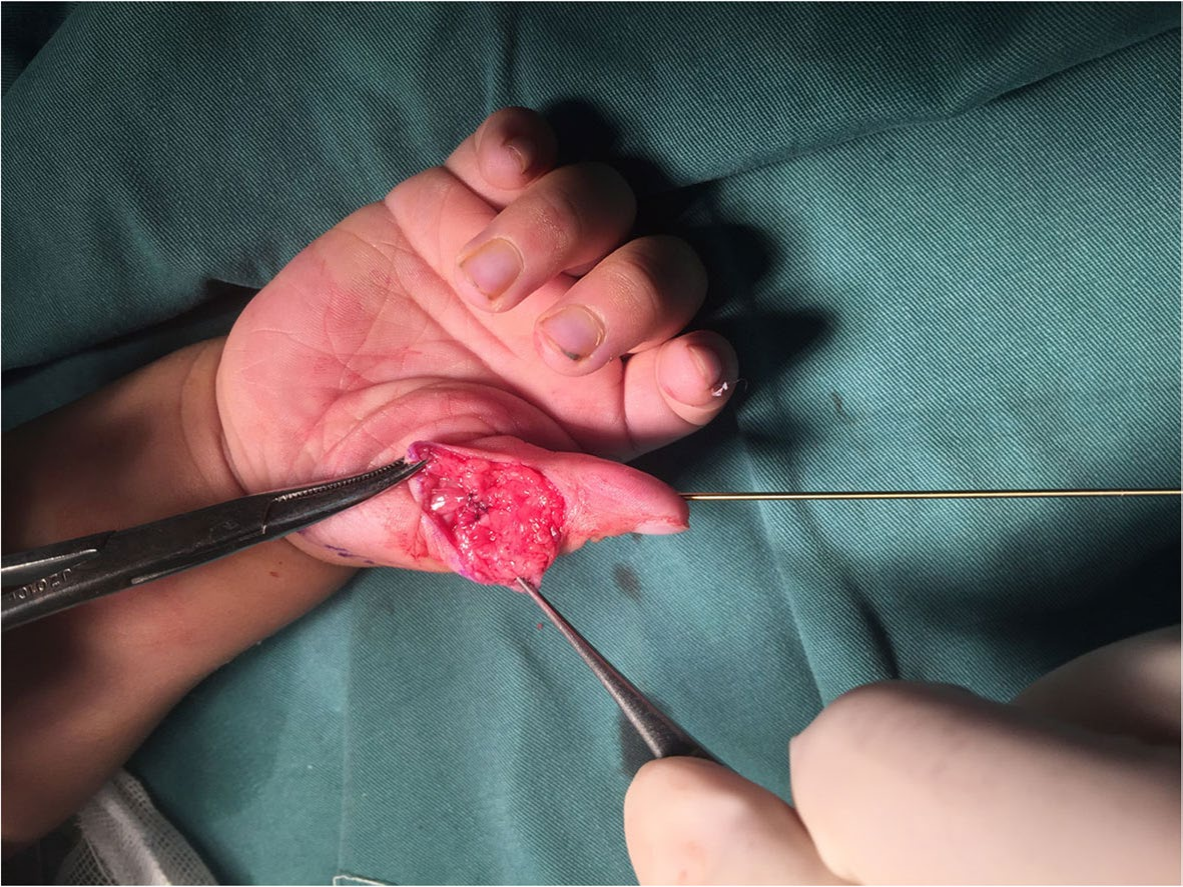
The preserved radial collateral ligament at MP joint and abductor pollicis brevis tendon were sutured to the radial side of the reconstructed thumb’s proximal phalanx

The preserved radial collateral ligament at MP joint and abductor pollicis brevis tendon were sutured to the radial side of the reconstructed thumb’s proximal phalanx, which could stabilize the MP joint and restore abduction (Fig. 7).

**Fig. 8:**
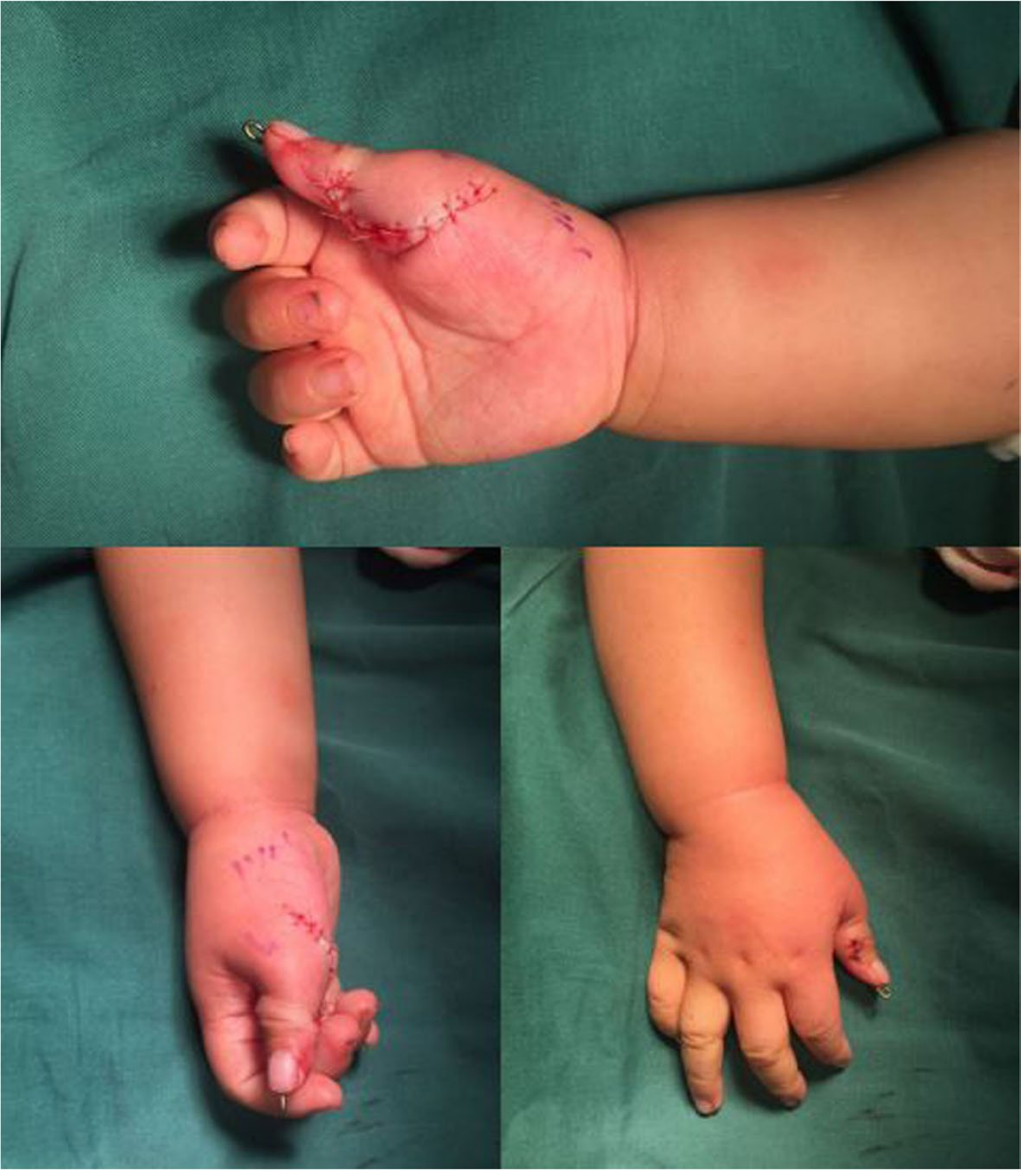
The skin incision is tailored for a smooth curvilinear scar and the wound is closed with an absorbable suture

The skin incision was tailored for a smooth curvilinear scar on the palmar side and the wound was closed with an absorbable suture (Fig. 8). A digital block was provided for postoperative analgesia and a long-arm cast was placed.

The cast and K wire were removed 4 weeks after surgery. Since then, active exercises were encouraged.

### Evaluation methods

The clinical results were evaluated using the Rotterdam assessment system (Table 1) for radial polydactyly [5]. Rotterdam assessment system included functional domain, appearance domain and patient-reported domain. Functional domain included active IP joint and MP joint flexion, IP joint and MP joint extension lag, IP and MP joint instability and Palmar abduction. A handheld goniometer was used in functional domain. Appearance of the scar, residual prominence at the amputation site, thumb size, pulp, and nail were separately quantified on a visual analog scale (VAS) score (0–100) ranging from “extremely ugly” to “perfectly normal looking” [4]. Patient-reported domain included pain and satisfaction. Pain and satisfaction were divided into four levels separately. Different levels correspond to different scores (Table 1).

**Table 1:** Rotterdam outcome assessment system for radial polydactyly

**Function**	Range	Points	**Appearance**	Range	Points
**Active IPJ + MPJ** **Flexion(°)**	≥ 130	3	**Scar (VAS)**	≥ 95	3
	111–129	2		86–94	2
	91–110	1		71–85	1
	≦90	0		≦70	0
**IPJ + MPJ** **Extension** **lag(°)**	≦20	2	**Prominence** **(VAS)**	≥ 95	3
	21–34	1		86–94	2
	≥ 35	0		66–85	1
				≦65	0
**MPJ instability** **(°)**	≦20	2	**Size (VAS)**	≥ 90	2
	21–29	1		76–89	1
	≥ 30	0		≦75	0
**IPJ instability** **(°)**	≦5	2	**Pulp (VAS)**	≥ 85	2
	6–9	1		76–84	1
	≥ 10	0		≦75	0
**Palmar** **Abduction** **(°)**	≥ 55	1	**Nail (VAS)**	≥ 85	1
	≦54	0		≦84	0
			**IPJ Deviation(°)**	≦5	2
				6–14	1
				≥ 15	0
			**MPJ deviation(°)**	≦10	1
				≥ 11	0
**Patient** **Reported**					
**Pain**	Never	3	**Satisfaction**	Maximal	3
	When cold	2		Reasonable	2
	With use	1		Moderate	1
	Constant	0		Dissatisfied	0
**Subtotal** **functional** **domain**					/10 points
**Subtotal** **appearance** **domain**					/14 points
**Subtotal** **Patient** **Reported** **domain**					/6 points
**Total outcome** **Score**					/30 points

The results of the Rotterdam assessment system were presented as mean (SD).

## Results

A total of 206 duplicated thumbs in 173 patients (92 males and 81 females) underwent surgical intervention in our institution between 2010 and 2019 by the same senior surgeon, different from the investigator. The mean follow–up period was 2.2 years (SD 1.5).

Table 2 showed the outcome results after resection and reconstruction surgery. According to Rotterdam system, the mean range of active IP and MP joint flexion was 110° (SD 32). Active IP and MP joint extension lags 26° (SD 20). Angulation for IP and MP joint instabilities were 3° (SD 3) and 11° (SD 5), relatively. Angulation for palmar abduction was 58° (SD 8). The mean functional domain score for all patients was 6.6 (SD 2.0).

**Table 2:** The outcome results of Rotterdam assessment after resection and reconstruction surgery for radial polydactyly Type IV-D

	Mean (SD)
Active IP joint and MP joint flexion (degrees)	110 (32)
IP joint and MP joint extension lag (degrees)	26 (20)
IP joint instability (degrees)	3 (3)
MP joint instability (degrees)	11 (5)
Palmar abduction (degrees)	58 (8)
**Functional domain score**	6.6 (2.0)
Scar	94 (16)
Prominence at amputation site	86 (20)
Size	75 (15)
Pulp	76 (15)
Nail	85 (18)
IP joint deviation (degrees)	5 (3)
MP joint deviation (degrees)	9 (6)
**Appearance domain score**	8.9 (2.8)
Patient-reported pain	2.1 (1.8)
Patient-reported satisfaction	2.5 (1.8)
**Patient-reported domain score**	4.5 (1.3)
**Rotterdan Score**	20 (1.7)

The mean appearance domain score was 8.9 (SD 2.8). The average parental satisfaction score was 2.5 (SD1.8) and the average pain score was 2.1 (SD 1.8). The mean patient-reported domain score was 4.5 (SD 1.3). The mean Rotterdam score was 20 (SD 1.7), equivalent to 67% of the full score.

Some degree of MP joint stiffness was common seen within two months after surgery in 74 patients. After motion exercises, joint stiffness was relieved. Tissue swelling occurred in 37 patients within two months after surgery. After two months, the swelling gradually vanished. No other surgical complications occurred.

There were 144 duplicated thumbs of patients, who did the operation when they were less than one year old. The mean Rotterdam score of those patients was 20.4 (SD 1.5)0.39 duplicated thumbs were corrected when the patients were 1–2 years old, whose average Rotterdam score was 20.0 (SD 1.1). The other 23 duplicated thumbs were corrected when the patients were more than 2 years old. The mean Rotterdam score was 17.5 (SD 2.0). There was no difference of Rotterdam score with surgery within 2 years (*p* > 0.05), while the postoperative score of patients over two years old was significantly lower than that of patients under two years old (*p* < 0.05).

## Discussion

Due to the complexity of the hand’s anatomy, thumb deformities are associated with a high incidence of postoperative deformity and dysfunction [7]. Regardless of which of the two thumbs is preserved in a Wassel IV-D type duplication, the IP and MP joints of the retained digit tend to deviate in opposite directions causing a zig-zag deformity [8]. Therefore, the reconstruction procedure included osteotomy and fixation, tendon and ligament reconstructions. The lack of any surgical procedure can be associated with secondary deformities [3]. The causes might be the mechanical imbalance of the thumb, skeletal remains or poor appearance with the increase of age.

The traditional incision for reconstructing one of the two thumbs was a racket shape, which resulted in a linear scar on the radial dorsal side of the thumb. The linear scar passed across the MP joint, which made patients feel uncomfortable. The linear scar was obviously seen because of the position. In the study, we designed two equal triangle flap incisions to make the scar on the palmar side, which was more hidden compared with the radial dorsal incision. The new incision formed a curvilinear scar across the MP joint. The curvilinear scar was smooth without traction. Different procedures (reshaped or wedge osteotomy) would be decided according to the angle of angular deformity, which made the angulations at the MP and IP joints realigned effectively. We combined objective examination with subjective assessment to evaluate the effect of the operation, including RAM, angulation, function, appearance, pain, and degree of satisfaction. The RAM of IP and MP joint flexion and extension, combined with the angulation for palmar abduction was suitable for patients to grasp objects. Angulations for IP and MP joint instabilities were both small, which indicated the IP and MP joint were stable after reconstruction. The mean appearance domain score was not low, which could be accepted by parents. The average pain score showed there were no other pains for most of patients except cold pain. The reason might be the process of sensory nerve reinnervation in local flap. It took time to reconstruct the temperature sense. The average parental satisfaction score showed the resection and reconstruction method was satisfying on the whole. The mean Rotterdam score was equivalent to 67% of the full score, which meant the operation method was basically accepted by the parents. As for the surgery time, the average Rotterdam score of patients with surgery within 2 years was significantly larger than that after 2 years old, which indicated severe deviation was more likely to occur after 2 years old and surgery was recommended under 2 years old.

As for the treatment of radial polydactyly Type IV-D, except resection and reconstruction procedure, the Bilhaut-Cloquet technique was another choice, which consisted of a central wedge resection of the duplication followed by fusion of the remaining parts [10]. The technique was described by Bilhaut in 1889 [2]. The drawbacks with the Bilhaut technique included nail deformity, thumb broadness, limitation of joint mobility and growth arrest.

Robert R carried out a matched comparative study of the Bilhaut procedure versus resection and reconstruction for treatment of radial polydactyly types II and IV [4]. The results showed unfavorable effects of the Bilhaut procedure on the postoperative appearance of the thumb compared reconstruction method. Thumb strength was less after the Bilhaut procedure compared with reconstruction. According to Rotterdam system assessment, our results coincided with the result of reconstruction method in Robert R’s study. A. Abid et al [1]. introduced a modified Bilhaut-Cloquet procedure which preserved the nail of the ulnar thumb duplicate totally. At the longest follow-up, the mean ranges of motion were 25° (range: 20°—35°) in flexion at MP joint and 10° (range: 5°—20°) in flexion at IP joint. The RAM of IP and MP joint was less than our result. Among them, two cases had 10° and 15° residual fixed flexion deformity relatively. In all the cases, range of motion in extension was normal at MP joint. No axial deviation was noted. The MP and IP joints were deemed stable in all cases. In general, the thumb after modified Bilhaut-Cloquet procedure was not as flexible as that after our surgery.

There were some drawbacks in the study. There was no control group in the study, such as contralateral thumb’s condition, so statistical analysis could not be carried out. As for the evaluation tool, although the Rotterdam system assessment covered objective and subjective aspects, including functional, appearance and patient-reported domains, it was a lack of a grading of scores. In the future research, we will add Tada score for the postoperative evaluation, which could categorize the results as good, fair or poor. The follow-up period was relatively short for functional measure and we would prolong the follow-up time in the further research.

## Conclusion

Resection and reconstruction method with two equal triangle flap incisions was a recommended treatment for radial polydactyly Type IV-D.

## Data Availability

The datasets used and analysed during the current study were available from the corresponding author on reasonable request.
